# Management of Adverse Effects in Testosterone Replacement Therapy

**DOI:** 10.1590/S1677-5538.IBJU.2025.9904

**Published:** 2025-01-31

**Authors:** Basheer Basheer, Vishal Ila, Rodrigo Barros, Francesco Mesquita, Leonardo Seligra Lopes, Victor Fernandes Negris Lima, Luciano A. Favorito, Ranjith Ramasamy

**Affiliations:** 1 Jumeirah American Clinic Dubai UAE Jumeirah American Clinic, Dubai, UAE;; 2 University of Miami Miller School of Medicine Miami FL USA Miller School of Medicine, University of Miami, Miami, FL, USA;; 3 Universidade Federal Fluminense Hospital Universitário Antônio Pedro Serviço de Urologia Niterói RJ Brasil Serviço de Urologia, Hospital Universitário Antônio Pedro - Universidade Federal Fluminense - UFF, Niterói, RJ, Brasil;; 4 Faculdade de Medicina Departamento de Urologia São José do Rio Preto SP Brasil Departamento de Urologia, Faculdade de Medicina (FAMERP/FUNFARME), São José do Rio Preto, SP; Brasil;; 5 Centro Universitário Faculdade de Medicina ABC Santo André SP Brasil Centro Universitário, Faculdade de Medicina ABC - FMABC, Santo André, SP, Brasil;; 6 Universidade Federal do Espírito Santo Departamento de Urologia Vitória Brasil Departamento de Urologia, Universidade Federal do Espírito Santo - UFES, Vitória, Brasil;; 7 Universidade do Estado do Rio de Janeiro Unidade de Pesquisa Urogenital Rio de Janeiro RJ Brasil Unidade de Pesquisa Urogenital, Universidade do Estado do Rio de Janeiro – UERJ, Rio de Janeiro, RJ, Brasil

**Keywords:** Testosterone, adverse effects [Subheading], therapy [Subheading]

## Abstract

**Purpose:**

This narrative review aims to provide the most updated knowledge regarding the treatment of adverse effects secondary to testosterone replacement therapy (TRT), such as gynecomastia, cardiovascular and hematologic risks, prostate health risk, and liver dysfunction risks.

**Materials and Methods:**

An extensive literature review was conducted, incorporating guidelines from the American Urological Association and the Endocrine Society. The studies determined common adverse effects and their most common methods of management.

**Results:**

TRT improves the quality of life, sexual function, and mood in hypogonadal men. Possible adverse effects associated with TRT include increased estrogen levels and gynecomastia, which are usually managed with aromatase inhibitors and tamoxifen. Cardiovascular risks from TRT include hypertension and erythrocytosis, which mandate periodic hematocrit and blood pressure monitoring; therapeutic phlebotomy is indicated if the hematocrit exceeds 52%. No significant concern regarding prostate cancer has been observed in the closely monitored patient. However, TRT should not be administered to individuals with active evidence of untreated prostate cancer, except under rare circumstances such as active surveillance for very low-risk disease. Older oral forms of TRT can affect liver function; therefore, transdermal, newer oral forms and injectables are generally favored in men with a history of liver disease.

**Conclusions:**

Monitoring and management of adverse effects are critical to maximize benefit and minimize the risks of TRT. Ongoing research will further elucidate the safety of TRT while advancing evidence-based practices in managing its associated adverse effects. Effective patient education and counseling are also essential to improve compliance and treatment outcomes.

## INTRODUCTION

Hypogonadism, or testosterone deficiency, is a condition that affects approximately 30 million men worldwide, with its prevalence increasing with age (1). Characterized by low serum testosterone levels, associated symptoms of hypogonadism include fatigue, decreased libido, erectile dysfunction, and mood disturbances (2). Signs of testosterone deficiency may also include reduced muscle mass, increased body fat, and diminished bone density. Some underlying illnesses that can exacerbate testosterone deficiency are obesity, metabolic syndrome, and various chronic illnesses (3). Testosterone replacement therapy (TRT) is designed to restore normal testosterone levels, potentially reversing some of these symptoms and improving overall health. In addition to TRT, other treatments aimed at improving testosterone levels are being explored. Varicocele repair, when clinically indicated, has shown potential to increase endogenous testosterone production (4). Additionally, selective estrogen receptor modulators like clomiphene citrate and the use of human chorionic gonadotropin (hCG) have emerged as alternatives, especially for men seeking to preserve fertility while increasing testosterone levels (5, 6). These approaches offer options for tailored management in men with low testosterone, particularly when traditional replacement therapy may not be ideal.

TRT can improve the quality of life, sexual function, and mood in hypogonadal men (7). Administering TRT requires several potential side effects to be considered: the likelihood of increased estrogen levels, gynecomastia, cardiovascular issues, prostate problems, and hematologic changes ^(8–10)^. Recent literature contains new information about the safety of TRT and strategies for minimizing adverse effects (11). This narrative review enables an understanding of these factors, allowing for treatment optimization and safety in hypogonadal men receiving TRT. Therefore, complications related to testosterone abuse, as well as changes in male infertility, were not included in this study.

### Increased Estrogen Levels and Gynecomastia Management

Gynecomastia has been observed when high levels of estrogen build up in men receiving TRT. Serum estradiol levels above 60 pg/mL may cause gynecomastia(8). Management strategies include the use of aromatase inhibitors such as anastrozole, which effectively reduces estrogen levels when the threshold for serum estradiol is exceeded (7). Dosage adjustment including lower doses of testosterone or even a switch to less aromatizing formulations such as testosterone undecanoate, will minimize estrogenic side effects (2). A wait-and-observe approach may be appropriate in cases of gynecomastia appearing without increased estrogen, as the condition can sometimes resolve spontaneously (9). Symptomatic gynecomastia can be treated with low-dose tamoxifen to alleviate breast tissue enlargement (12). In men with normal estrogen levels who have undergone at least 12 months of observation and experience psychological distress and cosmetic concerns, elective plastic surgery could be considered (12).

### Prostate Health

There is some controversy linking TRT with prostate health. Several meta-analyses utilizing contemporary studies have established that carefully monitored TRT has no significant risk for prostate cancer (13). In addition, TRT is contraindicated in patients with a history of untreated prostate cancer or active cancer. Conversely, benign prostatic hyperplasia (BPH) symptoms can be exacerbated with testosterone treatment.

Management strategies include regular monitoring of PSA levels, particularly in older patients or those with any history of prostate issues (3). In most cases, men above 50 years old should be followed up yearly with PSA levels. Younger men with risk factors of prostate cancer should have their PSA checked every 2-4 years (14). Normal PSA levels is usually below 4 ng/mL, though this may be age- dependent; for example, a man between 40-49 can have normal level up to 2.5 ng/mL, while men aged 70 and above may have a higher acceptable level. Men receiving TRT will have an associated increase of 0.30 ng/mL in PSA levels, with older men experiencing a greater increase of 0.43 ng/mL (15). If PSA levels increase, further investigation is warranted, including a repeat PSA test, a digital rectal exam and possibly imaging studies such as magnetic resonance imaging (MRI) to assess any abnormalities. In cases of TRT in patients who have been treated for prostate cancer, for example, post-radiotherapy or surgery, any rise in PSA level warrants a review of the treatment plan which may include discontinuing TRT and further oncological assessment (14, 16). Counseling regarding possible prostate-related risks associated with the initiation of therapy is imperative (3, 16). The presence of urinary symptoms among patients should be monitored when managing BPH (17, 18). For patients with symptomatic BPH, stopping TRT and definitively treating BPH with surgery may be required if medical management fails.

### Cardiovascular and Hematologic Risks

Cardiovascular risks associated with TRT have been well discussed. Some studies indicate that it has been proven to improve lipid profiles as well as endothelial function (19). On the contrary, it is also well known to raise blood pressure and thrombotic risks especially in older men with already pre-existing cardiovascular disease. Erythrocytosis or increased red blood cell mass is one of the common adverse effects of TRT that heighten thrombotic risks. As pointed out by Kohn et al., one of the side effects of TRT is an increase in hematocrit levels and thus this must be carefully monitored (20). Increased hematocrit is associated with a high risk of major adverse cardiovascular events (MACE) particularly if significantly higher from baseline. The hematocrit significantly increases with TRT irrespective of the formulation, with intramuscular testosterone enanthate/cypionate causing the most significant increase of 4.0%. For oral testosterone undecanoate, the increase is roughly 4.3%, but the patch and nasal gel preparations result in much more modest increases. Compared in one of the studies of intranasal versus intramuscular therapies, intramuscular injections significantly increased hematocrit from 42.7% to 46.6%, while there was no significant change with the intranasal gel. Such findings reiterate the variability of hematocrit response with different testosterone formulations and point out the need for monitoring hematologic parameters as a way to prevent cardiovascular risks, most especially in patients with already existing cardiovascular conditions (21, 22).

Management strategies should include referral to a cardiologist and should include regular monitoring of blood pressure and lipid profile in the high-risk group (23). It is also necessary to check the hematocrit regularly, especially in the first year of treatment, every 3-6 months in the beginning and annually thereafter (1).

Ory et al. (24) tried to find the unsafe hematocrit threshold for men receiving TRT and determine whether secondary polycythemia causes an increased risk of cardiovascular complications. They performed a retrospective cohort study from a database of 74 million people including two groups of men with low testosterone who received TRT and subsequently either did or did not develop polycythemia and compared 5,842 men in each group. Polycythemia was defined as a hematocrit above 52%, according to the American Urological Association (AUA) guideline definition. The primary outcome was incidence of MACE and venous thromboembolic events (VTE) in the first year of TRT. The authors found that men on TRT who developed secondary polycythemia had a higher incidence risk of MACE and VET than men who did not develop polycythemia (24). This cutoff can guide our clinical practice, and we can tell patients undergoing TRT that they are at a higher cardiovascular risk if their hematocrit reaches or exceeds 52% during the first year of therapy (25). Therefore, when the hematocrit exceeds these levels, it may be a sign that the patient requires therapeutic phlebotomy to prevent thrombotic complications (11).

Dose adjustment has to be made based on hematocrit; the marked erythrocytosis has to be avoided. El-Khatib et al. (26), indicated that reduction in the dose of testosterone injections coupled with an increase in their frequency of administration should help manage hematocrit and, therefore, reduce the risk of MACE. For instance, splitting a 100 mg dose into two or three smaller doses throughout the week may effectively maintain testosterone levels while minimizing the potential for elevated hematocrit and associated cardiovascular events.

Routine monitoring may also prevent extra cardiovascular risk because of fluid retention (3). Fluid retention may increase blood pressure; hence, patients should be counseled on dietary sodium restriction, monitoring fluid input, and blood pressure monitoring to prevent this complication effectively. It also allows the surveillance of the lipid profile for cardiovascular safety in testosterone-treated patients, since the treatment with testosterone may decrease HDL and probably increase LDL, more conditions that should be closely monitored in order to prevent or minimize the vascular changes resulting from changes in the level of lipids. However, various RCTs and meta-analyses conducted have suggested that routine monitoring of lipid profiles is probably not necessary in all patients. Indeed, a recent study by Calof et al. showed that adverse events from TRT in middle-aged and older men do not support the consistent monitoring of lipids (27). In another systematic review and meta-analysis, Haddad et al. concluded that TT does not result in overall significant changes in cardiovascular risk profiles among this population (28). According to TRAVERSE study, even men with hypogonadism and preexisting or a high risk of cardiovascular disease, TRT was non-inferior to placebo with respect to the incidence of MACE (29). Thus, these findings would suggest that although monitoring may be important, in many instances it may not be required, especially when the total cardiovascular risk is low.

### Liver Function

Certain formulations of testosterone, particularly oral routes, carry a risk for hepatic toxicity. Monitoring liver function and regularly testing for liver enzymes is recommended, especially among patients with a history of liver conditions, particularly in men receiving older oral forms of TRT (30). The preferred formulations are transdermal and injectable testosterone due to the lower risk of hepatic toxicity associated with these modes of administration (31). Goldstein et al., 2024 indicate that newer oral formulations, transdermal and injectable testosterone, have a significantly lower risk of hepatic toxicity compared to older oral formulations. The authors indicate that among oral formulations, the newer formulation of oral testosterone undecanoate (Tlando™, Kyzatrex™, and Jatenzo™) are relatively safer regarding liver effects as compared to the older methylated testosterone formulations (Andriol™) (32). The mechanism of absorption plays an important role in this difference. Newer oral preparations, which include testosterone undecanoate, utilize a different route of absorption via the lymphatic system, thereby avoiding major first-pass metabolism of the drug in the liver. Older oral formulations, such as Andriol™, increase liver function toxicity because of first-pass metabolism, exposing the liver to higher concentrations of the drug. Because this pathway minimizes hepatic exposure, liver enzymes are present in low amounts in order to minimize the risk for hepatic toxicity (32). This shift in formulation choice can alleviate the risks associated with liver function, highlighting the importance of selecting an appropriate TRT for each patient.

### Acne

Acne is a common consequence of TRT because sebaceous gland activity is increased, which is associated with the androgenic effects of the skin. Topical therapies include the use of retinoids or benzoyl peroxide, that can effectively manage mild to moderate acne. In cases of severe acne, oral antibiotics such as, minocycline, or isotretinoin may be indicated (33). It is also very important that patients are educated on proper skin care to reduce the incidence of acne outbreaks, and referral to the dermatologist should be considered for those with persistent or severe cases.

### Sleep Apnea

Obstructive sleep apnea (OSA) issues should be monitored among the patients, particularly when symptoms involve loud snoring or excessive daytime sleepiness (16, 34). OSA must be screened by using the STOP-BANG questionnaire prior to initiating TRT (35). If the screening of the patient comes out positive, then OSA should be appropriately treated. This becomes important because, unless treated, OSA may result in intermittent hypoxia that could lead to increased hematocrit levels as a result of the body adapting to a state of decreased oxygenation. Observation and management of these conditions are important to minimize adverse effects associated with TRT.

**Figure 1:**
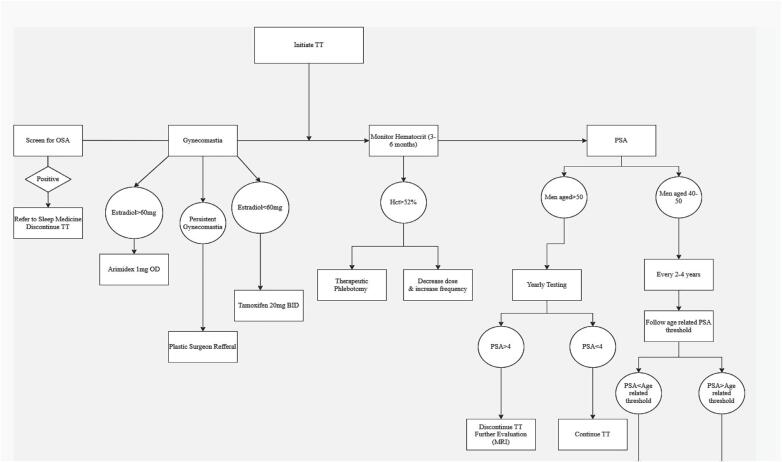
Treatment algorithm of complications after testosterone replacement therapy.

Treatment algorithm of complications after TRT is present in Figure 1.

### Patient Education and Informed Consent

Patient education is a vital component of managing adverse effects in TRT. Recent guidelines emphasize the importance of counseling patients on the benefits and risks of TRT (3, 34, 36). Shared decision-making may improve adherence and satisfaction with treatment, allowing patients to make informed choices about their health.

Well-educated patients are more likely to adhere to therapeutic regimens and become more active participants in their care. Effective communication regarding possible adverse effects, monitoring regimens, and lifestyle changes can empower patients and enhance the effectiveness of treatment. Additionally, supporting telemedicine can provide patients with convenient access to healthcare professionals for ongoing education and management. If adverse effects arise, patients should be encouraged to report these issues promptly through telehealth channels, allowing adjustments to their treatment plan.

## CONCLUSIONS

TRT is beneficial for patients with testosterone deficiency but monitoring and management of adverse effects are highly relevant to ensure safety and efficacy. Ongoing research will further delineate the safety profile of TRT and evidence-based practices for monitoring and managing adverse effects. With appropriate monitoring protocols, regular follow-ups, and prioritized patient education, healthcare providers can ensure that risks associated with TRT are minimized.
